# Association between resilience and advance care planning during the COVID-19 pandemic in Japan: a nationwide cross-sectional study

**DOI:** 10.1038/s41598-023-28663-4

**Published:** 2023-01-25

**Authors:** Jun Miyashita, Taro Takeshima, Kazuhira Maehara, Sugihiro Hamaguchi, Shunichi Fukuhara

**Affiliations:** 1 Department of General Medicine, Shirakawa Satellite for Teaching And Research (STAR), Fukushima Medical University, 2-1, Toyochikamiyajiro, Shirakawa, Fukushima 961-0005 Japan; 2 Center for University-Wide Education, School of Health and Social Services, Saitama Prefectural University, Saitama, Japan; 3 Department of General Internal Medicine, Fukushima Medical University Hospital, Fukushima, Fukushima Japan; 4 Section of Clinical Epidemiology, Department of Community Medicine, Graduate School of Medicine, Kyoto University, Kyoto, Japan; 5 Department of Health Policy and Management, Johns Hopkins Bloomberg School of Public Health, Baltimore, MD USA

**Keywords:** Human behaviour, Outcomes research, Palliative care

## Abstract

Advance care planning (ACP) is essential for end-of-life care, especially during the novel coronavirus disease 2019 (COVID-19) pandemic, and resilience is critical to deal with pandemic-related stressors. Therefore, we investigated the association between resilience ability and ACP discussions during the COVID-19 pandemic. A nationwide web-based survey was conducted in July 2021 in Japan. We analyzed the respondents’ ability to bounce back from stress (bouncing-back ability), positive stress coping (e.g., “seeking social support”, “planning”), and negative stress coping (e.g., “avoidance”, “alcohol and drug use”) in relation to ACP discussions using multivariable logistic regression models. In total, 2000 responses (86% participation rate) were received (mean age: 51.8 ± 16.7). Normal and high bouncing-back ability (adjusted odds ratio [AOR]: 1.69, 95% CI: 1.03–2.79; AOR: 2.07, 95% CI: 1.18–3.65, respectively) were significantly associated with the occurrence of ACP discussions. Seeking social support and planning were significantly associated with ACP discussions, whereas avoidance and alcohol and drug use were not. Both bouncing-back ability and positive stress coping were significantly associated with the occurrence of ACP discussions during the COVID-19 pandemic in Japan. These findings could be useful for aiding health-care providers involved in ACP discussions during the COVID-19 pandemic.

## Introduction

Advance care planning (ACP) is a process that facilitates adult understanding and the sharing of personal values, life goals, and preferences in regard to future medical care^1,2^. Previous studies have reported the effects of patient-centered ACP discussions on the quality of end-of-life care, emotional trauma in families, and the utilization of health-care services^3–5^. Discussions about ACP have been more critical for end-of-life care in aged societies such as Japan, where the aged population is expected to reach 30% by 2025^6^. However, the prevalence of ACP discussions in Japan has been reported to be 35–40%^7,8^, which is substantially lower than that in the United States (67%)^9^ and Canada (52%)^10^. Previous studies have also reported factors specific to Japan that hinder the prevalence of ACP, such as family-centered decision-making^11^ and high-context culture^2^, in addition to physicians’ reluctance to engage in ACP^12,13^.

Discussions about ACP have been considered more essential during the pandemic caused by the novel coronavirus (severe acute respiratory syndrome coronavirus 2 [SARS-CoV-2]) disease of 2019 (COVID-19)^14,15^. During the COVID-19 pandemic, there have been places around the world where scarce life-sustaining treatments could not be provided to all patients in need. In such situations, “rationing” decisions had to be made, which were incredibly challenging to all health-care providers and patients involved^16^. ACP discussions could reduce the need to ration limited life-sustaining treatments by identifying patients who would not want to receive them if affected by severe COVID-19 pneumonia^14^. However, ACP discussions should not be used as triage allocation decisions, but rather, as processes where adults develop individualized care plans^14,15^. In addition, the medical conditions of patients with COVID-19 might suddenly deteriorate. Therefore, it is important for adults of any age or with any health condition to discuss ACP ahead of the possibility of health deterioration and before facing a health crisis to ensure that their personal values, life goals, and preferences for medical and long-term care are known^14,15^. Therefore, especially during the COVID-19 pandemic, meaningful ACP discussions should be required at an appropriate time.

During the pandemic, most people around the world have lived their life with limited daily activities through quarantine strategies ranging from social distancing to various degrees of lockdowns. A considerable proportion of people in this situation have experienced moderate to severe anxiety and depression^17^. Pandemic-related worry or anxiety has provoked stress^18^ and been associated with deteriorated mental health^19,20^. Therefore, resilience is essential for individuals exposed to many stressors related to the COVID-19 pandemic. Resilience has been defined in various ways as an outcome characterized by particular patterns of functional behavior despite associated risks, or as a dynamic process of adaptation to a risk setting that involves interaction between a range of risk and protective factors from the individual to the social^21^. Resilience at the individual level, defined as the ability to bounce back from^22^ and adapt positively to stress^23^, has been reported to be associated with a lower level of stress and anxiety during the COVID-19 pandemic among adults^24^ and health-care providers^25^. Previous studies have reported that mastery, a kind of motivation for being resilient, is associated with health outcomes, including pain control in patients with cancer^26^ and cardiometabolic health^27^. In addition, resilience ability has been shown to be associated with health behaviors such as smoking^28^ and binge drinking^29^. Therefore, resilience ability might be essential for lowering stress, obtaining good health outcomes, and engaging in health behaviors during the current situation characterized by the COVID-19 pandemic.

ACP could be conceptualized as a set of health behaviors with key constructs of perceived susceptibility, self-efficacy, and the barriers to and benefits of changing one’s behavior, because it is a set of activities that patients participate in to prepare for deteriorating health and end-of-life^30^. Therefore, resilience ability might be a catalyst for discussions in advance about whether people would want to receive certain types of medical care upon being diagnosed with COVID-19 or at their future end-of-life. Previous studies have reported the effects of the resilience ability of health-care providers, especially nurses, on ACP practice^31^. However, to our knowledge, no quantitative study has examined the association between resilience ability and ACP discussions among the general population. We hypothesize that resilience ability (bouncing-back ability and positive stress coping) would be associated with ACP discussions among Japanese adults during the COVID-19 pandemic. If such an association were to be identified, health-care providers could help promote ACP discussions based on the ability of patients to bounce back or their type of stress coping.

Therefore, in this study, we investigated whether bouncing-back ability and various types of stress coping were associated with the occurrence of ACP discussions. Additionally, we investigated whether bouncing-back ability mediated between stress coping and the occurrence of ACP discussions with family members, health-care providers, or both, using nationwide survey data.

## Methods

### Study design, setting, and participants

A nationwide survey was conducted from July 8 to 16, 2021. This was a period when the number of COVID-19 cases had just started to increase again during Japan’s fifth wave, the surge driven by the delta variant of SARS-CoV-2. The survey was a closed, voluntary, and web-based survey with 31 questionnaire items that used an e-mail registration system to prevent duplicate entries. The primary investigator, JM, and one co-author, SF, created the questionnaire draft and revised it after review by other co-authors. Next, the questionnaire was pretested through interviews with three patients to ensure that the questionnaire items were understandable. The final version of the questionnaire was then developed by pilot testing on 10 patients (see Supplementary text 1 “English-translated questionnaire”). The web-based questionnaire was distributed by an independent research company (Nippon Research Center). From a panel comprising about 1,400,000 residents throughout Japan registered at the Nippon Research Center, the sample was drawn and designed to represent the general Japanese population at the time of the national census in 2015 by using quota sampling. Quotas were set concerning age, sex, and region by dividing all of Japan into five areas (“Hokkaido and Tohoku”; “Kanto”; “Toukai, Koushinetsu, and Hokuriku”; “Kansai”; and “Chugoku, Shikoku, and Kyuushuu”). As for the sample size calculation, we assumed that the risk ratio of having ACP discussions between respondents with low and adequate resilience was 0.6 based on previous studies about ACP in Japan^8,32^. Because no data were available about the prevalence of those discussing ACP during the COVID-19 pandemic, we estimated the proportion of those discussing ACP during the COVID-19 pandemic to be 15–20%, assuming that about half of those who had discussed ACP before the start of the COVID-19 pandemic discussed ACP during the pandemic. With these assumptions, to achieve a 90% power with an alpha of 0.05, a maximum of 1962 samples (low-resilience group: 393, adequate-resilience group: 1569) was required. Therefore, samples were drawn until reaching 2000 quota samples. The respondents ranged in age from 20 to 84 years.

### Outcomes

Questionnaire items regarding ACP discussions during the COVID-19 pandemic were developed. First, before answering any of the items, the respondents were asked to read the following explanation regarding ACP: “Advance care planning is the process of making the wishes of individual patients known to persons close to them in regard to the health-care interventions, including life-sustaining treatments, they would wish to receive if they were to become injured or very ill^33^”. After reading the explanation, the respondents were asked whether they had ever discussed ACP with family members (Q1) or health-care providers (Q2). Those selecting “yes” to either Q1 or Q2 were asked when they had such discussions (Q3). They selected their response from the following alternatives: “Before the start of the COVID-19 pandemic, but not after (A1),” “Not before the start of the COVID-19 pandemic, but after (A2),” or “Both before and after the start of the COVID-19 pandemic (A3).” Those selecting “yes” to either Q1 or Q2 and selecting either “A2” or “A3” to Q3 were defined as those who had “ACP discussions during the COVID-19 pandemic”. Second, we developed questionnaire items regarding what they discussed in regard to ACP during the COVID-19 pandemic. They were asked to select answers from the following multiple-choice alternatives: “Ways of living in their future life”, “Wishes for health-care interventions if diagnosed with COVID-19”, “Wishes for health-care interventions at end-of-life”, and “Surrogates expressing patients’ wishes for health-care interventions if they were unable to express their own”.

### Exposure

The main exposure variable was resilience, a concept that has been defined in several ways. We focused on resilience ability at an individual level, defined according to previous studies as bouncing-back ability and positive stress coping^23,34^, because we wanted to measure how people in the COVID-19 era bounced back from and coped with stressors or adversities related to the pandemic.

First, we measured bouncing-back ability using the Japanese version of the Brief Resilience Scale (BRS)^35^, which assesses resilience in its original and most basic meaning, i.e., the ability to bounce back, and is uniquely and more directly related to health outcomes than are other components of resilience^34^. The scale demonstrated good convergent and predictive validities, as evidenced by its positive associations with social relations and optimism and its negative associations with perceived stress, anxiety, degression negative affect, and physical symptoms^34,36^. Regarding reliability, Smith et al.^34^ reported an internal consistency of 0.91, and Cronbach’s alpha in the present study was 0.89. The BRS is composed of six five-point items. For example, item 1 (a positively worded item) is “I tend to bounce back quickly after hard times”, and item 2 (a negatively worded item) is “I have a hard time making it through stressful events”. Respondents were asked the degree to which they agreed with each of the items based on the following alternatives: “Strongly disagree”, “Disagree”, “Neutral”, “Agree”, or “Strongly agree”. The score was an equally weighed sum of the six items using normal coding of items 1, 3, and 5, and reverse coding of items 2, 4, and 6, with total scores ranging from 6 to 30. We developed a three-step categorical variable, with the cutoff between low and normal bouncing-back ability defined as the 25th percentile of the total score and the cutoff between normal and high defined as the 75th percentile. In addition, we developed a binary variable by using the cutoff of the 25th percentile (low vs. adequate resilience).

Second, we measured the following four types of stress coping: “Encouraging each other and talking with family or friends (seeking social support)”, “Formulating a strategy for a problem (planning)”, “Avoiding thinking about a problem (avoidance)”, and “Using alcohol or drugs to calm down (alcohol and drug use)”. Respondents were asked how often they engaged in each of these stress-coping methods in their daily life during the COVID-19 pandemic based on the following alternatives: “Not at all”, “Rarely”, “Sometimes”, “Often”, or “Always”.

### Covariates

We also investigated the sociodemographic covariates related to ACP discussions reported in previous studies: age^37,38^, sex^37,38^, marital status^39^, annual income^40^, education level^40^, comorbidities^41^, experiences of caregivers for family members^8^, and religious beliefs^32^. We also measured the following clinically relevant covariates related to the COVID-19 pandemic: COVID-19 literacy, media exposure about COVID-19, stress and anxiety in relation to COVID-19, COVID-19 vaccination status, and incidence of COVID-19. COVID-19 literacy was measured using ten questionnaire items regarding the symptoms, prevention, and treatment of COVID-19, in reference to previous studies^42,43^. In the present study, Cronbach’s alpha was 0.85. Based on additional previous studies^44,45^, media exposure in relation to COVID-19 was measured by total time per day spent engaging with COVID-19-related information obtained from the following five types of media: television, newspaper, radio, online news, and social network services. In the present study, Cronbach’s alpha was 0.77. Referring to previous studies^46,47^, we measured stress and anxiety in relation to COVID-19 using the following two questions: “How much stress do you feel about the COVID-19 pandemic?” and “How afraid are you of being diagnosed with COVID-19?” The respondents were asked to answer using the following five-point scale: “1, not at all”; “2, rarely”; “3, sometimes”; “4, often”; or “5, always”’. We developed a binary variable of “stress and anxiety in relation to COVID-19” by using the total scores for these two questions with a cutoff of the median value (low vs. high).

### Statistical analyses

We used Student’s *t*-tests or chi-squared tests for bivariate analyses and developed multivariable logistic regression models^48^. In the primary analysis (Model 1), the three-step categorical variable of bouncing-back ability was used as the main exposure variable and “ACP discussions during the COVID-19 pandemic” was used for the outcome variable. We also performed secondary analyses in which stress coping (seeking social support, planning, avoidance, and alcohol and drug use) was used for the main exposure variable, with “ACP discussions during the COVID-19 pandemic” as the outcome variables in Models 2–5, respectively. A test of linear trends across degrees of bouncing-back ability and stress coping was used to evaluate the linearity of the relationship in each model. These multivariable logistic regression models (Models 1–5) were adjusted for age, sex, marital status, education level, annual income, comorbidities, caregiving experience, religious beliefs, COVID-19 literacy, media exposure about COVID-19, stress and anxiety in relation to COVID-19, COVID-19 vaccination status, and incidence of COVID-19 (See Supplementary Fig. 1 “Directed acyclic graph for Model 1”). The age variable was dealt with as a categorical variable according to previous studies^8,32^. In addition, because the scores for COVID-19 literacy had a left-skewed distribution and were not linearly related to the log odds of the response variable, a four-category variable with the quartiles as cutoffs was used in the logistic regression models. The variance inflation factor was used to examine multicollinearity. There were few missing values, so we did not use any imputation methods for the analyses.

In addition, we performed a regression-based approach for causal mediation analyses^49^ with binary exposure (frequency of stress coping: “not at all” vs. “rarely/sometimes/often/always”), mediator (low vs. adequate bouncing-back ability), and outcome (occurrence of ACP) to estimate the natural direct and indirect effects of stress coping on the occurrence of ACP discussions through the bouncing-back ability measured by the BRS. We adjusted for the covariates significantly associated with ACP discussions during the COVID-19 pandemic in Model 1. Two-sided* P* values less than 0.05 were considered significant. We used Stata/IC v.15 (College Station, TX) for the statistical analyses, with the Stata PARAMED macro used for causal mediation analyses^50^.

### Ethics approval and consent to participate

The study protocol was approved by the institutional review board of Shirakawa Kosei General Hospital (HAKURIN21-004). This study was conducted in accordance with the Declaration of Helsinki and the Ethical Guidelines for Medical and Health Research Involving Human Subjects^51^. An informed consent was obtained from all participants. Before participating in the survey, all respondents were informed that they were under no obligation to answer any questions. Completing the questionnaire was regarded as consent to participate.

## Results

### Participants’ characteristics

In total, 2000 responses were received (mean age ± SD, 51.8 ± 16.7 years). The participation rate (calculated as the number of those who filled in the first survey page divided by the number of visitors to the first page of the survey^52^) was 86%. Among the respondents, 979 (49%) were men. The median BRS score was 18 (interquartile range, 15–22). Therefore, we developed a three-step categorical variable of bouncing-back ability with the following scores: 6–14 (low), 15–22 (normal), and 23–30 (high). In total, 451 (22.6%), 1105 (55.3%), and 444 (22.2%) respondents had low, normal, and high bouncing-back ability, respectively. In addition, 187 (9.4%) respondents had discussed ACP during the COVID-19 pandemic, and 67 (3.6%) had discussed health-care interventions in the case they were diagnosed with COVID-19. In the present survey, 35.9% of the respondents had received at least one dose of a COVID-19 vaccine (vs. 33.3% in the Japanese population based on data released by the Japanese Prime Minister’s office on 16 July 2021^53^; Table 1).

**Table 1:** Respondents’ sociodemographic characteristics and factors regarding COVID-19.

Sociodemographic characteristics and factors regarding COVID-19	Total sample	Bouncing-back ability		*P*	Missing
		Low	Adequate		
		6–14	15–30		
	N = 2000	n = 451	n = 1549		0
Men, n (%)	979 (49.0)	172 (38.1)	807 (52.1)	< 0.001^a^	0
Age (years), mean (SD)	51.8 (16.7)	45.7 (14.5)	53.6 (16.9)	< 0.001^b^	0
Marital status, n (%)				< 0.001^a^	0
Married	1116 (55.8)	191 (42.4)	925 (59.7)		
Single	670 (33.5)	216 (47.9)	454 (29.3)		
Divorced	132 (6.6)	32 (7.1)	100 (6.5)		
Bereaved	82 (4.1)	12 (2.7)	70 (4.5)		
Education level > 12 years (vs. ≤ 12), n (%)	1400 (70.2)	311 (69.0)	1089 (70.5)	0.52^a^	5
Annual income (USD), n (%)				0.07^a^	0
< $30,000	525 (26.3)	135 (29.9)	390 (25.2)		
$30,000 to < $70,000	951 (47.6)	212 (47.0)	739 (47.7)		
≥ $70,000	524 (26.2)	104 (23.1)	420 (27.1)		
Comorbidities^c^, median (IQR)	0 (0–1)			0.001^a^	0
Without diseases, n (*%*)	1116 (55.8)	239 (53.0)	877 (56.6)		
With one disease, n (*%*)	481 (24.1)	137 (30.4)	344 (22.2)		
With two or more diseases, n (*%*)	403 (20.2)	75 (16.6)	328 (21.2)		
Family caregiving experience^d^ (vs. no experience), n (%)	539 (27.0)	108 (24.0)	431 (27.8)	0.10^a^	0
Religious beliefs^e^ (vs. no belief), n (%)	625 (31.3)	121 (26.8)	504 (32.5)	0.02^a^	0
COVID-19 literacy (0–10 points), median (IQR)	8 (6–9)			0.59^a^	0
7 or less out of 10 points, n (%)	514 (25.7)	105 (23.3)	409 (26.4)		
8 out of 10 points, n (%)	731 (36.6)	173 (38.4)	558 (36.0)		
9 out of 10 points, n (%)	483 (24.2)	111 (24.6)	372 (24.0)		
10 out of 10 points, n (%)	272 (13.6)	62 (13.8)	210 (13.6)		
Media exposure about COVID-19 (minutes/day), median (IQR)	65.7 (20–134.6)			0.33^a^	0
Lowest quartile (≥ 0 to < 20 min), n (%)	476 (23.8)	107 (23.7)	369 (23.8)		0
2nd quartile (≥ 20 to < 66 min), n (%)	527 (26.4)	129 (28.6)	398 (25.7)		
3rd quartile (≥ 66 to < 135 min), n (%)	497 (24.9)	116 (25.7)	381 (24.6)		
Highest quartile (≥ 135 min), n (%)	500 (25.0)	99 (22.0)	401 (25.9)		
COVID-19 incidence^f^, n (%)	24 (1.2)	3 (0.67)	21 (1.4)	0.24^a^	0
COVID-19 vaccination^g^, n (%)	717 (35.9)	85 (18.9)	632 (40.8)	< 0.001^a^	0
High stress/anxiety in relation to COVID-19, n (%)	660 (33.0)	223 (49.5)	437 (28.2)	< 0.001^a^	0
ACP discussions during the COVID-19 pandemic, n (%)	187 (9.4)	23 (5.1)	164 (10.6)	< 0.001^a^	0
Ways of living in their future lives	116 (5.8)	12 (2.7)	104 (6.7)	0.001^a^	0
Health-care interventions if diagnosed with COVID-19	67 (3.6)	7 (1.6)	60 (3.9)	0.02^a^	0
Health-care interventions at end-of-life	84 (4.2)	10 (2.2)	74 (4.8)	0.02^a^	0
Surrogates	55 (2.8)	8 (1.8)	47 (3.0)	0.15^a^	0

*IQR* interquartile range, *ACP* advance care planning.

^a^Chi-square test.

^b^Student’s *t*-test.

^c^Comorbidities were defined as the sum of the number of the following diseases or medical conditions: hypertension, hypercholesterolemia, diabetes mellitus, cerebrovascular disease, cardiac disease, respiratory disease, gastrointestinal disease, renal disease, urologic disease, hematologic disease, orthopedic disease, rheumatologic disease, neurological and psychiatric disease, endocrine disease, and neoplasm.

^d^The question to the participants: “Do you have experience as a family caregiver?”.

^e^The question to the participants: “Do you have any religious beliefs or affiliations?”.

^f^The question to the participants: “Have you received a positive result from a polymerase chain reaction (PCR) or antigen test for COVID-19?”.

^g^The question to the participants: “Have you been vaccinated against COVID-19 at least once?”.

### Association between bouncing-back ability and ACP discussions

Respondents with normal (adjusted odds ratio [AOR]: 1.69, 95% CI: 1.03–2.79) and high bouncing-back ability (AOR: 2.07, 95% CI: 1.18–3.65) had significantly higher odds of having had ACP discussions than those with low bouncing-back ability (*P* for trend = 0.03). In addition, female, older, religious respondents with more comorbidities who had family caregiving experience and had been infected with COVID-19 had significantly higher odds than their counterparts (Table 2). No significant multicollinearity was found in the model (maximum value of variance inflation factors: 2.12).

**Table 2:** Association between bouncing-back ability and advance care planning discussions during the COVID-19 pandemic (model 1).

Sociodemographic characteristics and factors regarding COVID-19	AOR, 95% CI
Bouncing-back ability (Brief Resilience Scale)^a^	
Normal, 15–22 (vs. low, 6–14)	1.69, 1.03–2.79*
High, 23–30 (vs. low, 6–14)	2.07, 1.18–3.65*
Men (vs. women)	0.66, 0.47–0.93*
Age (years)	
55–64 (vs. 20–54)	1.56, 0.96–2.52
65–74 (vs. 20–54)	1.51, 0.88–2.59
75–84 (vs. 20–54)	2.30, 1.16–4.54*
Marital status	
Married (vs. single)	1.22, 0.76–1.94
Divorced (vs. single)	0.56, 0.23–1.34
Bereaved (vs. single)	0.78, 0.35–1.75
Education level, > 12 years (vs. ≤ 12 years)	0.98, 0.68–1.41
Annual income (USD)	
≥ $30,000 to < $70,000 (vs. < $30,000)	1.00, 0.66–1.52
≥ $70,000 (vs. < $30,000)	1.33, 0.82–2.16
Comorbidities^a^	
With one disease (vs. without disease)	1.34, 0.87–2.04
With two or more diseases (vs. without disease)	2.21, 1.47–3.32***
Family caregiving experience (vs. no experience)	1.99, 1.42–2.79***
Religious beliefs (vs. no religious beliefs)	2.07, 1.47–2.92***
COVID-19 literacy	
8 points (vs. 7 or less points)	0.66, 0.43–1.03
9 points (vs. 7 or less points)	0.52, 0.32–0.86*
10 points (vs. 7 or less points)	0.72, 0.42–1.23
Media exposure about COVID-19 (minutes)^a^	
≥ 20 to < 66 (vs. ≥ 0 to < 20)	1.10, 0.64–1.88
≥ 66 to < 135 (vs. ≥ 0 to < 20)	1.43, 0.85–2.40
≥ 135 (vs. ≥ 0 to < 20)	1.55, 0.94–2.58
COVID-19 incidence (vs. no incidence)	4.31, 1.61–11.52**
COVID-19 vaccination (vs. no vaccination)	1.18, 0.80–1.75
High stress/anxiety in relation to COVID-19 (vs. low stress/anxiety)	1.24, 0.87–1.76

*AOR* adjusted odds ratio.

*P < 0.05; **P < 0.01; ***P < 0.001.

^a^*P* for trend was < 0.05.

### Association between types of stress coping and ACP discussions

**Figure 1:**
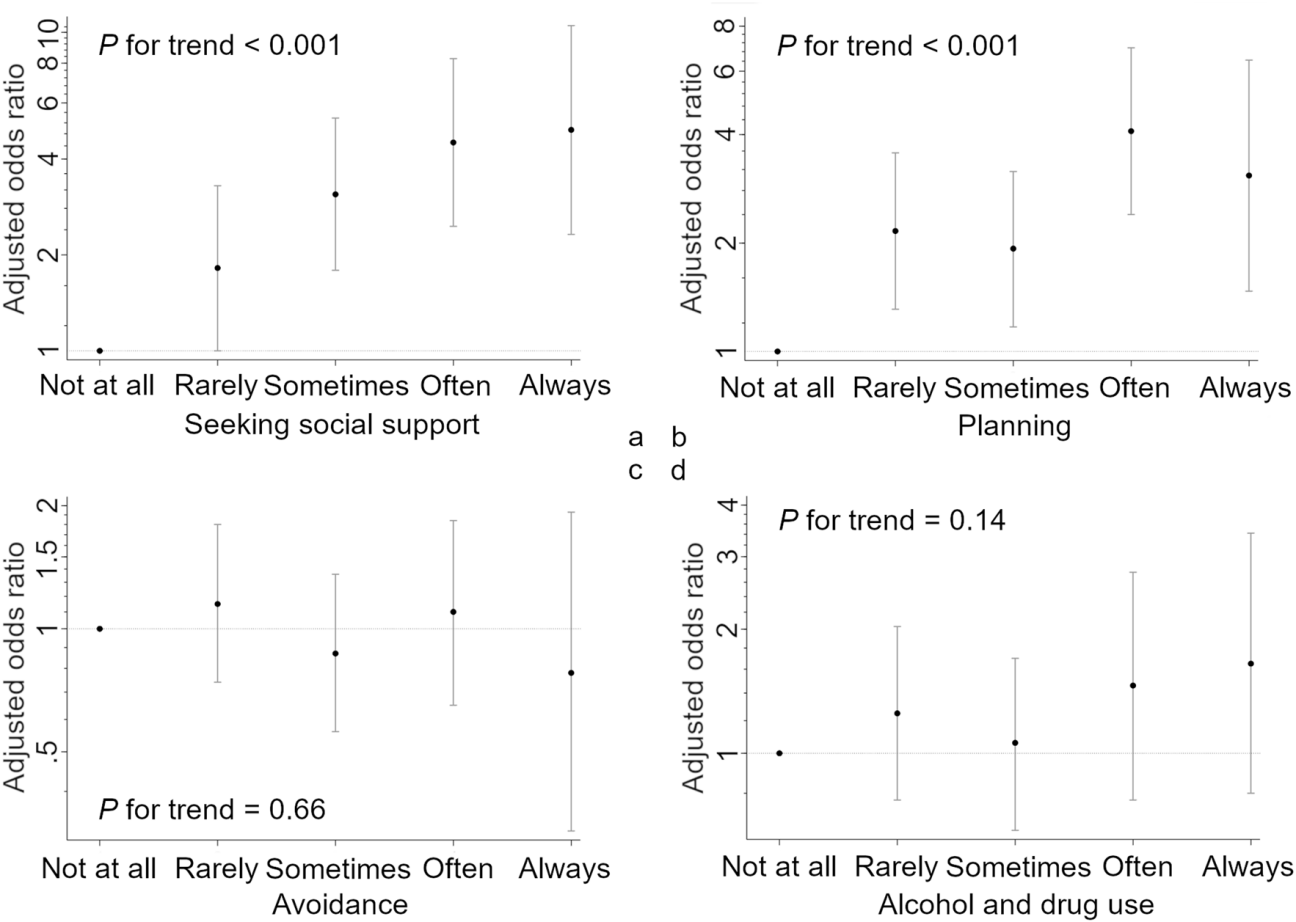
Odds ratios for the occurrence of advance care planning discussions by type of stress coping. (**a–d**) The results of the multivariable logistic regression models (Models 2–5), respectively. In each model, stress coping (seeking social support, planning, avoidance, and alcohol and drug use) was used as the main exposure variables, with “advance care planning discussions during the COVID-19 pandemic” as the outcome variable. All models were adjusted for age, sex, marital status, education level, annual income, comorbidities, caregiving experience, religious beliefs, COVID-19 literacy, media exposure about COVID-19, stress and anxiety in relation to COVID-19, COVID-19 vaccination status, and COVID-19 incidence as covariates.

**Figure 2:**
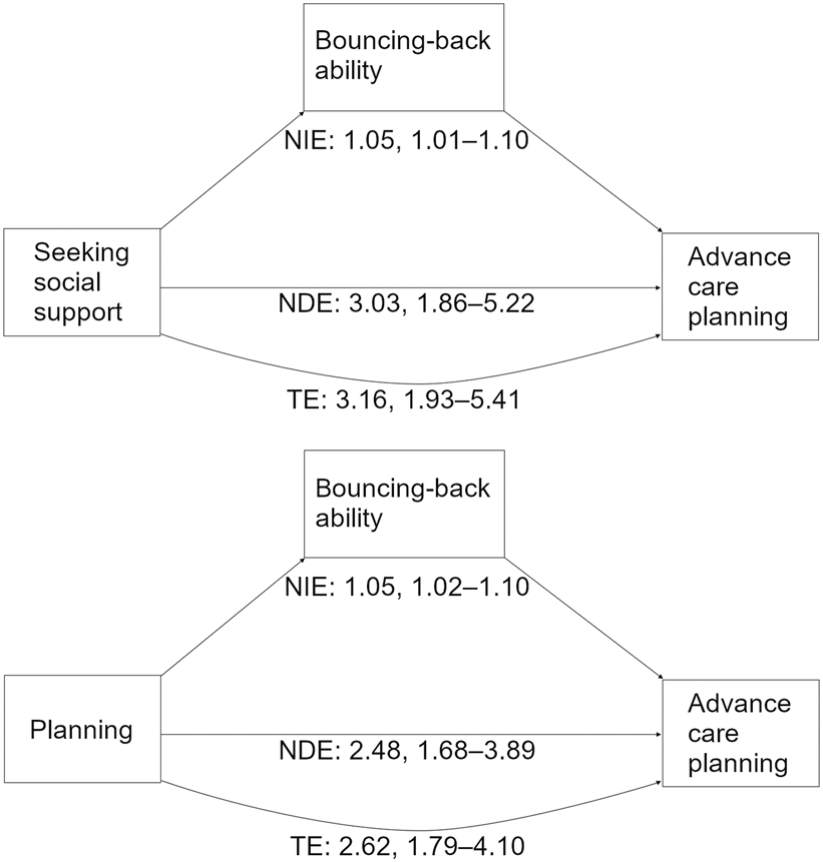
Mediation process and results regarding stress coping on advance care planning discussions through bouncing-back ability. *NIE* natural indirect effect, *NDE* natural direct effect, *TE* total effect. The upper and lower diagrams in the figure show models regarding stress coping related to seeking social support and planning on advance care planning discussions through bouncing-back ability, respectively. The numbers in the figure indicate ORs and bias-corrected bootstrapped 95% CIs representing the natural direct and indirect effects and the total effect. The models included age, sex, comorbidities, caregiving experience, religious beliefs, and COVID-19 incidence as covariates.

Regarding stress coping, Fig. 1 shows that seeking social support and planning were significantly associated with the occurrence of ACP discussions, whereas avoidance and alcohol and drug use were not (see Supplementary Tables 1–4). We therefore investigated whether bouncing-back ability mediated between the occurrence of ACP and seeking social support and planning. The results indicated that bouncing-back ability was significantly associated with seeking social support and planning. However, causal mediation analyses revealed that the odds ratios for the natural indirect effects of seeking social support and planning on ACP discussions mediating bouncing-back ability were 1.05 (95% bootstrapped CI, 1.01–1.10) and 1.05 (95% bootstrapped CI, 1.02–1.10), respectively; thus, bouncing-back ability was barely attributable to mediation between these types of stress coping and ACP discussions (Fig. 2).

## Discussion

### Summary of the main results

The results of this study indicated that Japanese adults with higher bouncing-back ability who engaged in positive stress coping (seeking social support and planning) were significantly more likely to have ACP discussions during the COVID-19 pandemic. In addition, bouncing-back ability barely helped mediate between positive stress coping and ACP discussions.

### Interpretation of the results

According to Smith et al.^34^, resources for resilience such as positive stress coping (e.g., seeking social support and planning) may facilitate the ability to bounce back, which, in turn, may have a more direct relationship with positive health outcomes. Therefore, we hypothesized that seeking social support and planning would be associated with ACP discussions via bouncing-back ability. However, our results did not support this hypothesis regarding the indirect effect of positive stress coping on ACP discussions through bouncing-back ability. Therefore, three resilience components—bouncing-back ability, planning, and seeking social support—might be independently associated with ACP discussions.

Regarding bouncing-back ability in relation to ACP discussions, a previous study^54^ provided a possible explanation for an underlying mechanism. Individuals with bouncing-back ability tend to have a more realistic perspective; they come to understand that although they cannot control everything, they have the power to influence what happens. Therefore, they can act proactively when faced with adversity. In-line with this mechanism, adults with bouncing-back ability consider that they might not be able to control their health in the future during the COVID-19 pandemic, so they may choose to prepare for their future life and medical needs. Therefore, preparedness for the future among adults with bouncing-back ability could facilitate ACP discussions.

According to the theory of stress and coping by Lazarus et al.^55,56^, there are two stress-coping types: a problem-focused form (e.g., problem-solving, confrontive coping) used when exposed to external stressors considered changeable, and an emotion-focused form (e.g., avoidance, distancing) used among those appraised as requiring acceptance. Therefore, stress coping associated with planning could enable individuals to consider that stressors related to the COVID-19 pandemic are changeable, so they might want to prepare for their future life and medical needs.

Several previous studies support our findings regarding stress coping by seeking social support during the COVID-19 pandemic^8,33,39,57^. Individuals with better family relationships in Japan are more likely to have experiences involving informal care for family members^33^. These experiences would encourage them to develop their own preferences for their future life and medical needs^8^. In addition, good family relationships enable individuals to discuss the potentially difficult topic of end-of-life care with their family members^39^.

### Clinical implications

These findings suggest that three resilience components—bouncing-back ability, planning, and seeking social support—are associated with ACP discussions via their own unique pathways. However, at the same time, the distinct pathways from the three components to ACP discussions might be mediated by a common factor, that is, “preparedness for the future.” A previous study^58^ reported that those who value individual control over the dying process were more likely to engage in ACP discussions than were those who did not. Thus, all three resilience components could be sources of power to encourage individuals to have more control over the dying process and to prepare proactively for their future life or medical needs in the case of being diagnosed with COVID-19 or requiring end-of-life care.

### Strengths and limitations

In this nationwide cross-sectional study, we found for the first time that both bouncing-back ability and positive stress coping were significantly associated with the occurrence of ACP discussions during the COVID-19 pandemic. However, this study had several limitations. First, we focused only on bouncing-back ability and positive stress coping; we did not measure other components of resilience or resilience resources such as the personal characteristics of optimism, sense of humor, patience^59^, equanimity, or perseverance^60^. These unmeasured components could affect our results regarding the measured components of resilience in relation to ACP discussions. Second, although we adjusted the multivariable regression models for socioeconomic factors associated with ACP and possibly relevant COVID-19-related factors, we could not deny the possible involvement of some unmeasured covariates, such as the relationship between individuals and the health-care providers involved in their care and treatments. Third, causal relationships could not be inferred because of the cross-sectional design of the study. However, reverse causality might be unlikely because in this study, resilience was defined as a trait or ability. Fourth, because of the cross-sectional design, the temporal ordering of variables in the mediation analyses might be unclear, although the hypotheses we examined in our mediation analyses were mentioned in a previous study^34^. Fifth, because this was a web-based survey, it is possible that we could not reach some of the target population who were not proficient at using the Internet, especially older people. Sixth, our sample size could be relatively small for a national survey, although sampling was performed based on a sample size calculation. Finally, it might be difficult to extrapolate our findings to other countries because our findings comprise only Japanese cultural aspects.

### Future directions

Among the three resilience components (i.e., bouncing-back ability, planning, and seeking social support) related to ACP discussions, bouncing-back ability and planning might be more important features than seeking social support during the COVID-19 pandemic. As most people in the world have been forced to live isolated daily lives because of quarantine policies in many countries, it might sometimes be difficult to seek social support. Therefore, bouncing-back ability and positive stress coping such as planning should be learned and strengthened throughout the COVID-19 pandemic. Further studies are needed to develop ACP promotion programs using interventions tailored on the level of bouncing-back ability and the stress-coping strategies that could encourage preparedness for the future.

## Conclusion

Both bouncing-back ability and positive stress coping were significantly associated with the occurrence of ACP discussions during the COVID-19 pandemic in Japan. These findings could help health-care providers become more involved in adult engagement in ACP discussions during the COVID-19 pandemic.

## Supplementary Information

Supplementary Information 1.

Supplementary Information 2.

## Data Availability

Miyashita had full access to all the data collected in the study and takes responsibility for the integrity of the data and the accuracy of the data analysis. All data generated or analyzed during this study are included in this published article.
