# Supplementary material for: Association between resilience and advance care planning during the COVID-19 pandemic in Japan: a nationwide cross-sectional study
Source: Sci Rep. 2023 Jan 25;13:1371. doi: 10.1038/s41598-023-28663-4 (PMC9876997; doi:10.1038/s41598-023-28663-4)

We would like to ask about your health

Q1 The following questions are about activities you might do during a typical day. Does your health now limit you in these activities? If so, how much?

	Yes, limited a lot		Yes, limited a little		No, not limited at all
a. Moderate activities, such as moving a table, pushing a vacuum cleaner, bowling, or playing golf	1	——	2	——	3
b. Climbing several flights of stairs	1	——	2	——	3

Q2 These questions are about how you feel and how things have been with you during the past four weeks. For each question, please give the one answer that comes closest to the way you have been feeling. How much of the time during the past four weeks...

	All of the time		Most of the time		Some of the time		A little of the time		None of the time
a. Have you felt calm and peaceful?	1	——	2	——	3	——	4	——	5
b. Have you felt downhearted and depressed?	1	——	2	——	3	——	4	——	5

Q3 Have you ever been told by a doctor, nurse, or other health-care providers that you have any of the following diseases? Please indicate your diseases by putting a cross in the following checkboxes.

	Yes ▼
Hypertension	□
Diabetes mellitus	□
Hyperlipidemia	□
Stroke	□
Myocardial infarction in the past year	□
Angina pectoris	□
Congestive heart failure or cardiomegaly	□
Eye diseases such as cataracts	□
Respiratory diseases such as asthma and pneumonia	□
Diseases of the digestive system, such as the stomach, intestines, and liver	□
Kidney diseases	□
Pancreatic diseases	□
Diseases of the urinary system, such as bladder and prostate	□
Blood diseases such as anemia	□
Diseases of the lower extremities, such as osteoarthritis and fractures	□
Rheumatic diseases such as rheumatoid arthritis and ankylosing spondylitis	□
Diseases causing low back pain, such as spinal stenosis	□
Shoulder diseases such as frozen shoulder	□
Atopic dermatitis	□
Nervous system diseases such as epilepsy	□
Mental illnesses such as depression	□
Diseases of the endocrine system, such as thyroid	□
Diseases of the female reproductive system, such as the uterus and ovaries	□
Cancer	□
Others ( )	□

Q4 How long have you had experiences caring for your family members at home or attending them in a hospital almost every day when they were ill? Please respond to choices by marking one number.

1. No experience	3. ≥ 1 and < 3 years of experience
2. < 1 year of experience	4. ≥ 3 years of experience

Q5 Please mark one number from each line. How often is someone available:

	None of the time	A little of the time	Some of the time	Most of the time	All of the time
to help you if you were confined to bed?	1	2	3	4	5
to take you to the doctor if you needed it?	1	2	3	4	5
to prepare your meals if you are unable to do it yourself?	1	2	3	4	5
to help with daily chores if you were sick?	1	2	3	4	5
to have a good time with?	1	2	3	4	5
to turn to for suggestions about how to deal with a personal problem?	1	2	3	4	5
who understands your problems?	1	2	3	4	5
to love and make you feel wanted?	1	2	3	4	5

Q6 Please respond to each item by marking one number per row.

	Strongly Disagree		Disagree		Neutral		Agree		Strongly Agree
1. I tend to bounce back quickly after hard times	1	——	2	——	3	——	4	——	5
2. I have a hard time making it through stressful events.	1	——	2	——	3	——	4	——	5
3. It does not take me long to recover from a stressful event.	1	——	2	——	3	——	4	——	5
4. It is hard for me to snap back when something bad happens.	1	——	2	——	3	——	4	——	5
5. I usually come through difficult times with little trouble.	1	——	2	——	3	——	4	——	5
6. I tend to take a long time to get over set-backs in my life.	1	——	2	——	3	——	4	——	5

Q7 Please respond to each knowledge item about coronavirus disease 2019 (COVID-19) by marking one number per row.

	Correct		Incorrect		Do not know
1. The main clinical symptoms of COVID-19 are fever, fatigue, dry cough, and myalgia.	1	——	2	——	3
2. There currently is no effective cure for COVID-19, but early symptomatic and supportive treatment can help most patients recover from infection.	1	——	2	——	3
3. Not all persons with COVID-19 will develop severe cases. Only those who are elderly and have chronic illnesses are more likely to be in severe cases.	1	——	2	——	3
4. The COVID-19 virus spreads via respiratory droplets of infected individuals.	1	——	2	——	3
5. Ordinary residents can wear general medical masks to prevent infection by the COVID-19 virus.	1	——	2	——	3
6. Children and young people do not need to take COVID-19 prophylaxis.	1	——	2	——	3
7. You should avoid going to crowded public places and not contracting COVID-19.	1	——	2	——	3
8. Isolating and treating people infected with COVID-19 is an effective way to reduce the spread of the virus	1	——	2	——	3
9. Persons who have been in close contact with a person infected with COVID-19 should immediately be quarantined in an appropriate place for an observation period.	1	——	2	——	3
10. Since the COVID-19 vaccines are messenger RNA, they can be incorporated into DNA and harm the human body.	1	——	2	——	3

Q8 Please respond to each perception item about COVID-19 by marking one number per row.

	not at all	rarely	sometimes	often	always
1. How much stress do you feel about the COVID-19 pandemic in the past month?	1 ——	2 ——	3 ——	4 ——	5
2. How afraid are you of being diagnosed with COVID-19 in the past month?	1 ——	2 ——	3 ——	4 ——	5

Q9 Please respond to each stress-coping item during the COVID-19 pandemic by marking one number per row.

	not at all	rarely	sometimes	often	always
1. Avoiding thinking about a problem	1 ——	2 ——	3 ——	4 ——	5
2. Using alcohol or drugs to calm down	1 ——	2 ——	3 ——	4 ——	5
3. Formulating a strategy for a problem	1 ——	2 ——	3 ——	4 ——	5
4. Encouraging each other and talking with family or friends	1 ——	2 ——	3 ——	4 ——	5

Q10 Have you ever been infected with COVID-19 and had a positive antigen or PCR test for COVID-19? Please respond to choices by marking one number.

1 Yes	2 No

Q11 If you respond to Yes in Q10, please respond to the question. What medical condition did you have when you were infected with COVID-19? Please respond to choices by marking one number.

1. No symptom<br>2. Cold symptoms such as fever, cough, and a sense of fatigue	3. Mild pneumonia<br>4. Pneumonia requiring oxygen inhalation	5. Severe pneumonia requiring intensive care such as ventilator

Q12 Have you been vaccinated against COVID-19 at least once? Please respond to choices by marking one number.

1 Yes	2 No

Q13 How much time do you spend a day getting information about COVID-19 from

media (television, newspapers, radio, online news on the Internet, and social network services such as Twitter and Facebook)? Please respond to each item by entering the number by the minute according to the example.

	weekdays		weekends
Example：1.5 hours on weekdays and 2 hours on weekends	90	……	120
1. Television		……	
2. Newspapers		……	
3. Radio		……	
4. Online news on the Internet		……	
5. Social network services such as Twitter and Facebook		……	

Q14 How much do you trust media (television, newspapers, radio, online news on the Internet, and social networking services such as Twitter and Facebook) regarding information about COVID-19 by marking one number per row?

	not at all	rarely	sometimes	often	always
1. Television	1 ——	2 ——	3 ——	4 ——	5
2. Newspapers	1 ——	2 ——	3 ——	4 ——	5
3. Radio	1 ——	2 ——	3 ——	4 ——	5
4. Online news on the Internet	1 ——	2 ——	3 ——	4 ——	5
5. Social network services such as Twitter and Facebook	1 ——	2 ——	3 ——	4 ——	5

We would like to ask you about advance care planning

Advance care planning (ACP) is the process of making the wishes of individual patients known to persons close to them regarding the health-care interventions, including life-sustaining treatments, they would wish to receive if they were to become injured or very ill; for example, infected with COVID-19.

Q15 Please respond to each item regarding ACP by marking one item per row.

1. I have discussed ACP with family members	1 Yes	2 No
2. I have discussed ACP health-care providers	1 Yes	2 No

If you selected "yes" to eihter Q15-1 or Q15-2, please respond to Q16, Q17, and Q18

If you selected "No" to both Q15-1 and Q15-2, please respond to Q19

Q16 When did you discuss ACP? Please respond to choices by marking one number.

1	2	3
Before the start of the COVID-19 pandemic, but not after	Not before the start of the COVID-19 pandemic, but after	Both before and after the start of the COVID-19 pandemic

Q17 What kind of content did you discuss? Please indicate all you have discussed by putting crosses in the following checkboxes.

	Yes ▼
1. Ways of living in their future life	☐
2. Wishes for health-care interventions if diagnosed with COVID-19	☐
3. Wishes for health-care interventions at end-of-life	☐
4. Surrogates expressing patients' wishes for health-care interventions if they were unable to express their own	☐

Q18 Do you record the contents of the discussion in documents or memos?

1. Yes	2. No

Please proceed to Q22

Q19 Do you feel you need to have discussions about ACP during the COVID-19 pandemic?

1. Yes	2. No

If you respond to No, please respond to Q20 and Q21

If you respond to No, please proceed to Q22

Q20 What kind of content do you want to discuss? Please indicate all you have discussed by putting crosses in the following checkboxes.

	Yes ▼
1. Ways of living in their future life	□
2. Wishes for health-care interventions if diagnosed with COVID-19	□
3. Wishes for health-care interventions at end-of-life	□
4. Surrogates expressing patients' wishes for health-care interventions if they were unable to express their own	□

Q21 Do you record the contents of the discussion in documents or memos?

1. Yes	2. No

Q22 Please assume that you become as follows in the future.

・ In the future, you become unconscious due to a severe illness or injury, with a very low possibility of recovery.

・ In the future, you will be unable to make decisions due to dementia.

In these cases, do you wish to receive life-sustaining treatment* to keep you alive? Please respond to choices by marking one item that applies to your current opinion. (Select only one choice)

**Life-sustaining treatment includes chest compressions and inotropic drugs when the heart stops, a ventilator when the patient can no longer breathe, and the placement of a feeding tube. These treatments can sustain life for a certain time (from a few days to several years), but there is no possibility of regaining consciousness.*

1. I want to receive life-sustaining treatments.
2. I do not want to receive life-sustaining treatments.
3. I leave my family members or those close to me to decide whether to receive life-sustaining treatments.
4. I cannot think about whether to receive life-sustaining treatments.

We would like to ask you about yourself

Q23 What is your date of birth? Year: ______ Month: ______

Q24 What is your gender?

1 Male	2 Female

Q25 Please select the one answer that best describes your current marital status.

1. Single	3. Separate	5. Bereaved
2. Married	4. Divorced	6. Other ( )

Q26 Who are you currently living with? (Select multiple choices)

1. Alone	4. with parents	7. with grandchildren
2. with a spouse	5. with siblings	8. with others
3. with children	6. with grandparents	( )

Q27 What is the annual income of your household? Please estimate the annual income of all members of the same household, including tax, wages/salaries, income from side jobs, pensions, remittances, and other income. (Select only one choice)

1. < \$30,000	4. \$70,000 to < \$100,000
2. \$30,000 to < \$50,000	5. \$100,000 to < \$120,000
3. \$50,000 to < \$70,000	6. ≥ \$120,000

Q28 Do you go to school now? If you are currently enrolled, please let us know the school, and if you are graduated, please let us know the last school you graduated from. (Select only one choice)

1. Elementary school/junior high school	5. University
2. High school	6. Graduate school
3. Professional training college	7. Other schools
4. Junior college	( )

Q29 Do you have any religious beliefs or affiliations? (Select only one choice)

1. Yes<br>2. My family has a religious affiliation	3. No

If you respond 1 or 2 in Q29, Please respond to Q30.

If you respond 3 in Q29, that concludes the questionnaire.

Q30 What are your religious beliefs or affiliations?

Q31 Do you feel that you are devout? (Select only one choice)

1. Very devot	3. A little devout
2. Somewhat devout	4. Not devout

Thank you very much for your cooperation.

Supplementary table 1. Association between Seeking Social Support and Advance Care Planning Discussions during the COVID-19 Pandemic (Model 2)

**Sociodemographic characteristics and factors regarding COVID-19**	**AOR, 95% CI**
Seeking social support[a]	
Rarely (vs. Not at all)	1.82, 1.00–3.30*
Sometimes (vs. Not at all)	3.09, 1.78–5.37***
Often (vs. Not at all)	4.48, 2.45–8.22***
Always (vs. Not at all)	4.94, 2.32–10.54***
Men (vs. women)	0.74, 0.53–1.05
Age (years)	
55–64 (vs. 20–54)	1.82, 1.11–2.97*
65–74 (vs. 20–54)	1.85, 1.08–3.18*
75–84 (vs. 20–54)	2.56, 1.29–5.07**
Marital status	
Married (vs. single)	1.03, 0.64–1.67
Divorced (vs. single)	0.50, 0.20–1.23
Bereaved (vs. single)	0.67, 0.29–1.54
Education level, > 12 years (vs. ≤ 12 years)	0.96, 0.66–1.38
Annual income (USD)	
≥ $30,000 to < $70,000 (vs. < $30,000)	0.95, 0.62–1.44
≥ $70,000 (vs. < $30,000)	1.32, 0.81–2.15
Comorbidities[a]	
With one disease (vs. without disease)	1.37, 0.90–2.10
With two or more diseases (vs. without disease)	2.35, 1.56–3.55***
Family caregiving experience (vs. no experience)	1.86, 1.32–2.63***
Religious beliefs (vs. no religious beliefs)	1.94, 1.37–2.75***
COVID-19 literacy	
8 points (vs. 7 or less points)	0.69, 0.44–1.07
9 points (vs. 7 or less points)	0.53, 0.32–0.88*
10 points (vs. 7 or less points)	0.72, 0.42–1.23
Media exposure about COVID-19 (minutes)	
≥ 20 to < 66 (vs. ≥ 0 to < 20)	1.03, 0.60–1.78
≥ 66 to < 135 (vs. ≥ 0 to < 20)	1.25, 0.74–2.10
≥ 135 (vs. ≥ 0 to < 20)	1.32, 0.79–2.21
COVID-19 incidence (vs. no incidence)	4.19, 1.50–11.66**
COVID-19 vaccination (vs. no vaccination)	1.12, 0.75–1.68
High stress/anxiety in relation to COVID-19 (vs. low stress/anxiety)	1.00, 0.70–1.43

AOR, adjusted odds ratio.
[a]*P* for trend was < 0.05.
**P* < 0.05; ***P* < 0.01; ****P* < 0.001.

Supplementary table 2. Association between Planning and Advance Care Planning Discussions during the COVID-19 Pandemic (Model 3)

**Sociodemographic characteristics and factors regarding COVID-19**	**AOR, 95% CI**
Planning[a]	
Rarely (vs. Not at all)	2.15, 1.31–3.55**
Sometimes (vs. Not at all)	1.92, 1.17–3.15*
Often (vs. Not at all)	4.06, 2.34–6.92***
Always (vs. Not at all)	3.05, 1.46–6.38**
Men (vs. women)	0.68, 0.48–0.95*
Age (years)	
55–64 (vs. 20–54)	1.64, 1.01–2.67*
65–74 (vs. 20–54)	1.67, 0.97–2.85
75–84 (vs. 20–54)	2.58, 1.32–5.07**
Marital status	
Married (vs. single)	1.26, 0.79–2.02
Divorced (vs. single)	0.60, 0.25–1.47
Bereaved (vs. single)	0.77, 0.34–1.74
Education level, > 12 years (vs. ≤ 12 years)	0.94, 0.65–1.35
Annual income (USD)	
≥ \$30,000 to < \$70,000 (vs. < \$30,000)	0.94, 0.62–1.43
≥ \$70,000 (vs. < \$30,000)	1.28, 0.79–2.09
Comorbidities[a]	
With one disease (vs. without disease)	1.32, 0.87–2.03
With two or more diseases (vs. without disease)	2.24, 1.49–3.38***
Family caregiving experience (vs. no experience)	1.92, 1.36–2.70***
Religious beliefs (vs. no religious beliefs)	2.08, 1.47–2.94***
COVID-19 literacy	
8 points (vs. 7 or less points)	0.69, 0.44–1.07
9 points (vs. 7 or less points)	0.52, 0.32–0.86*
10 points (vs. 7 or less points)	0.70, 0.41–1.20
Media exposure about COVID-19 (minutes)	
≥ 20 to < 66 (vs. ≥ 0 to < 20)	1.02, 0.59–1.76
≥ 66 to < 135 (vs. ≥ 0 to < 20)	1.32, 0.78–2.12
≥ 135 (vs. ≥ 0 to < 20)	1.36, 0.82–2.29
COVID-19 incidence (vs. no incidence)	3.93, 1.43–10.91**
COVID-19 vaccination (vs. no vaccination)	1.22, 0.81–1.82
High stress/anxiety in relation to COVID-19 (vs. low stress/anxiety)	1.08, 0.76–1.54

AOR, adjusted odds ratio.
[a]*P* for trend was < 0.05.
**P* < 0.05; ***P* < 0.01; ****P* < 0.001.

Supplementary table 3. Association between Avoidance and Advance Care Planning Discussions during the COVID-19 Pandemic (Model 4)

**Sociodemographic characteristics and factors regarding COVID-19**	**AOR, 95% CI**
Avoidance	
Rarely (vs. Not at all)	1.15, 0.74–1.80
Sometimes (vs. Not at all)	0.87, 0.56–1.36
Often (vs. Not at all)	1.09, 0.65–1.83
Always (vs. Not at all)	0.78, 0.32–1.92
Men (vs. women)	0.69, 0.49–0.97*
Age (years)	
55–64 (vs. 20–54)	1.64, 1.01–2.66*
65–74 (vs. 20–54)	1.72, 1.01–2.93*
75–84 (vs. 20–54)	2.73, 1.40–5.33**
Marital status	
Married (vs. single)	1.24, 0.78–1.98
Divorced (vs. single)	0.57, 0.24–1.37
Bereaved (vs. single)	0.79, 0.35–1.77
Education level, > 12 years (vs. ≤ 12 years)	1.01, 0.70–1.45
Annual income (USD)	
≥ $30,000 to < $70,000 (vs. < $30,000)	0.99, 0.65–1.50
≥ $70,000 (vs. < $30,000)	1.38, 0.85–2.23
Comorbidities[a]	
With one disease (vs. without disease)	1.28, 0.84–1.94
With two or more diseases (vs. without disease)	2.09, 1.39–3.13***
Family caregiving experience (vs. no experience)	1.96, 1.40–2.75***
Religious beliefs (vs. no religious beliefs)	2.06, 1.46–2.91***
COVID-19 literacy	
8 points (vs. 7 or less points)	0.66, 0.43–1.02
9 points (vs. 7 or less points)	0.52, 0.32–0.85*
10 points (vs. 7 or less points)	0.71, 0.42–1.21
Media exposure about COVID-19[a] (minutes)	
≥ 20 to < 66 (vs. ≥ 0 to < 20)	1.08, 0.63–1.85
≥ 66 to < 135 (vs. ≥ 0 to < 20)	1.39, 0.83–2.33
≥ 135 (vs. ≥ 0 to < 20)	1.56, 0.93–2.60
COVID-19 incidence (vs. no incidence)	4.72, 1.77–12.61**
COVID-19 vaccination (vs. no vaccination)	1.22, 0.82–1.82
High stress/anxiety in relation to COVID-19 (vs. low stress/anxiety)	1.15, 0.81–1.64

AOR, adjusted odds ratio.
[a]P for trend was < 0.05.
*$P < 0.05$; **$P < 0.01$; ***$P < 0.001$.

Supplementary table 4. Association between Alcohol and Drug Use and Advance Care Planning Discussions during the COVID-19 Pandemic (Model 5)

**Sociodemographic characteristics and factors regarding COVID-19**	**AOR, 95% CI**
Alcohol and drug use	
Rarely (vs. Not at all)	1.25, 0.77–2.30
Sometimes (vs. Not at all)	1.06, 0.65–1.70
Often (vs. Not at all)	1.46, 0.78–2.76
Always (vs. Not at all)	1.66, 0.80–3.42
Men (vs. women)	0.66, 0.46–0.94*
Age (years)	
55–64 (vs. 20–54)	1.70, 1.05–2.77*
65–74 (vs. 20–54)	1.79, 1.05–3.05*
75–84 (vs. 20–54)	2.97, 1.51–5.83**
Marital status	
Married (vs. single)	1.23, 0.77–1.96
Divorced (vs. single)	0.57, 0.24–1.39
Bereaved (vs. single)	0.79, 0.35–1.77
Education level, > 12 years (vs. ≤ 12 years)	1.01, 0.70–1.46
Annual income (USD)	
≥ \$30,000 to < \$70,000 (vs. < \$30,000)	1.01, 0.67–1.53
≥ \$70,000 (vs. < \$30,000)	1.38, 0.86–2.24
Comorbidities[a]	
With one disease (vs. without disease)	1.25, 0.82–1.91
With two or more diseases (vs. without disease)	2.06, 1.37–3.08***
Family caregiving experience (vs. no experience)	1.95, 1.39–2.73***
Religious beliefs (vs. no religious beliefs)	2.05, 1.45–2.89***
COVID-19 literacy	
8 points (vs. 7 or less points)	0.68, 0.44–1.06
9 points (vs. 7 or less points)	0.53, 0.32–0.87*
10 points (vs. 7 or less points)	0.74, 0.43–1.25
Media exposure about COVID-19[a] (minutes)	
≥ 20 to < 66 (vs. ≥ 0 to < 20)	1.09, 0.64–1.86
≥ 66 to < 135 (vs. ≥ 0 to < 20)	1.40, 0.84–2.35
≥ 135 (vs. ≥ 0 to < 20)	1.52, 0.91–2.53
COVID-19 incidence (vs. no incidence)	4.40, 1.64–11.80**
COVID-19 vaccination (vs. no vaccination)	1.23, 0.83–1.82
High stress/anxiety in relation to COVID-19 (vs. low stress/anxiety)	1.12, 0.79–1.59

AOR, adjusted odds ratio.
[a]*P* for trend was < 0.05.
**P* < 0.05; ***P* < 0.01; ****P* < 0.001.

Supplementary figure 1. Directed acyclic graph for Model 1

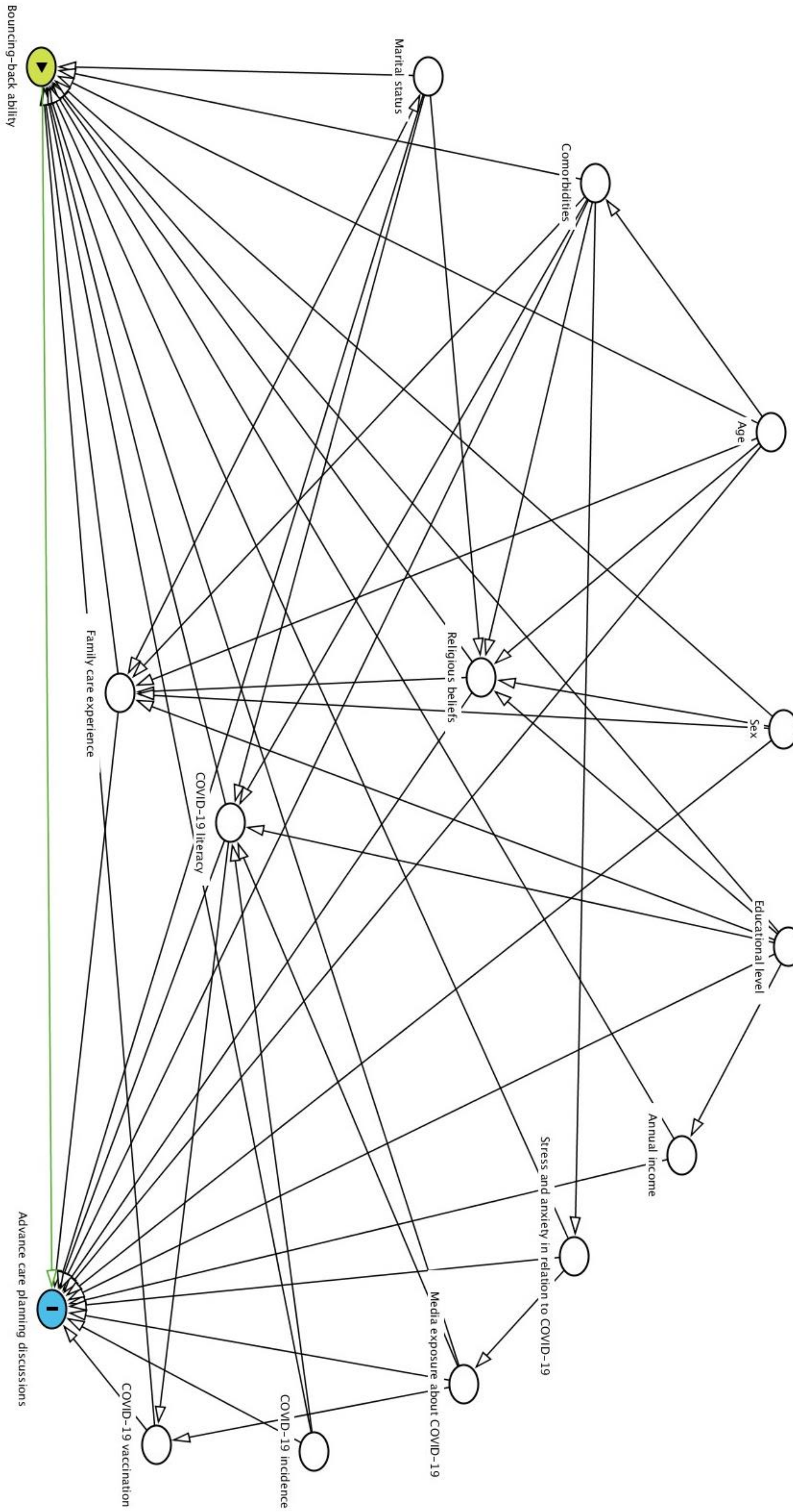

Supplement: Supplementary file 2 — Supplementary Information 2. [file 41598_2023_28663_MOESM2_ESM.pdf]
